# Diarrhoea Caused by Diffuse Metastatic Lobular Breast Cancer

**DOI:** 10.1155/2016/1785409

**Published:** 2016-05-22

**Authors:** Sjoerd F. Bakker, Willem Moolenaar, Marije M. van Santen, Mathijs P. Hendriks

**Affiliations:** ^1^Department of Gastroenterology and Hepatology, Northwest Clinics, Wilhelminalaan 12, 1815 JD Alkmaar, Netherlands; ^2^Department of Pathology, Northwest Clinics, Wilhelminalaan 12, 1815 JD Alkmaar, Netherlands; ^3^Department of Oncology, Northwest Clinics, Wilhelminalaan 12, 1815 JD Alkmaar, Netherlands

## Abstract

A 70-year-old woman with a history of lobular breast cancer presented to our Outpatient Clinic with diarrhoea for the past 3 years. Clinical examination and laboratory research were normal. Colonoscopy showed diffuse mild erythema and a decreased vascular pattern. Biopsies from the ascending colon, transverse colon, and descending colon showed metastases of lobular breast carcinoma. Although gastrointestinal metastases are rare in breast cancer, our case emphasizes the need for further diagnostic efforts in patients with gastrointestinal symptoms and a history of breast carcinoma.

## 1. Introduction

Breast cancer is the most frequently diagnosed cancer and the leading cause of cancer-related death among females worldwide. Common sites of breast cancer metastases include bones 85%, lung (15–25%), liver (40–50%), and brain (6–16%) [1]. Metastases to the gastrointestinal (GI) tract are uncommon (<1%) but are seen more frequently in lobular breast carcinoma than ductal carcinoma [2]. Few case reports describe breast cancer metastases to the colon [3]. Endoscopic findings of breast cancer metastases to the colon might appear as an obstructing mass, Crohn's disease, or polyps or simulate linitis plastica of the colon [3]. Here, we report a patient with diarrhoea with minimal abnormalities at colonoscopy which was caused by diffuse metastases of lobular breast carcinoma.

## 2. Case Report

A 70-year-old female presented with a 3-year history of diarrhoea. Her medical history consists of hypertension, atrial fibrillation, pacemaker placement, and left-sided mastectomy 4.5 years ago for a pT1cN3M0 oestrogen receptor (ER) 60% positive, progesterone receptor (PR) 90% positive, and Human Epidermal Growth Factor Receptor 2 (HER2) negative invasive lobular carcinoma. A Positron Emission Tomography-Computed Tomography showed no metastases. She received 45-gray chest wall radiation and adjuvant systemic therapy consisted of an aromatase-inhibitor (Anastrozole). No adjuvant chemotherapy was given.

**Figure 1:**
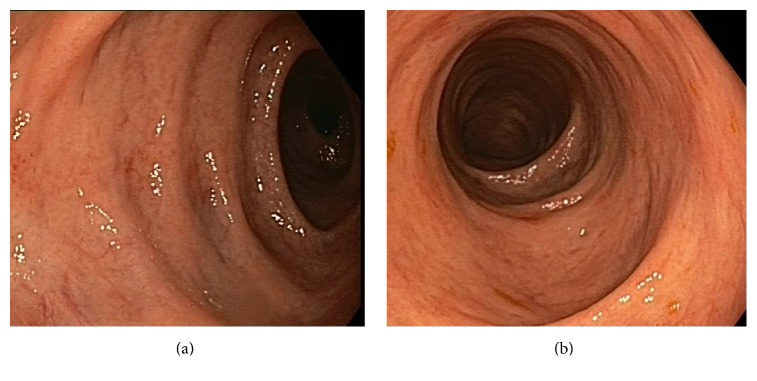
Macroscopic findings at colonoscopy. Ascending colon (a) and descending colon (b) are shown.

**Figure 2:**
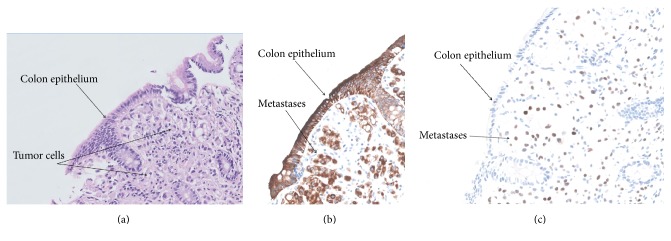
Histopathological examinations of colon biopsies reveal metastases of lobular breast carcinoma. (a) Haematoxylin-eosin (HE) staining, magnification 20x, (b) immunohistochemical staining for keratin, magnification 20x, and (c) immunohistochemical staining for oestrogen receptor, magnification 20x.

The patient was referred to the Department of Gastroenterology and Hepatology because of nonbloody diarrhoea with bowel movements 4–6 times a day. Clinical examination and laboratory results were normal. A colonoscopy was performed and showed minimal abnormalities: a decreased vascular pattern, diffuse mild erythema, tubular formation of the colon, and decreased haustra (Figure 1). Biopsies were taken at the level of the ascending colon, descending colon, and sigmoid. Microscopic examination revealed infiltration of the mucosal lamina propria by neoplastic cells in all biopsies, without disruption of the epithelial lining. The tumour cells resembled the cells in the original tumour and were typically those of a lobular carcinoma with a diffuse growth pattern and plasmacytoid and signet ring cell morphology (Figure 2). The phenotype of the neoplastic cells was coherent with the patient's previous lobular breast carcinoma: ER and PR were positive, Her2 was negative. Computed Tomography (CT) of the thorax and the abdomen showed no abnormalities of the colon and no lymphadenopathy in the abdomen. However, several lesions suspicious for lung metastases were observed. Second-line endocrine therapy with Tamoxifen was started and 8 weeks later she reported defaecation once daily. The diarrhoea had disappeared. Laboratory evaluation showed that the cancer antigen 15-3 level had dropped to 4879.0 kU/L compared to 7691.0 kU/L just before Tamoxifen was initiated.

## 3. Discussion

GI metastases of breast carcinoma are a rare entity. The stomach is the most common site of GI metastases of breast carcinoma [2]. Several cases of colonic metastases of breast cancer have been reviewed in the literature [3]. Endoscopic findings of breast cancer metastases to the colon might appear as an obstructing mass, Crohn's disease, or polyps or simulate linitis plastica of the colon [3].

In contrast, our case and another case from Zhang et al. [4] illustrate that endoscopic findings might be almost normal. Another case described the presence of linitis plastica of the colon caused by metastatic breast carcinoma [5]. However, in that case, abdomen CT showed marked thickness of the bowel wall and contrast enhancement of the colon. CT scan of our patient lacked these abnormalities of the colon, suggesting that colonoscopy with biopsies is crucial in these patients with diarrhoea to establish this diagnosis. In addition, irrespective of the past history, unexplained diarrhoea warrants colonoscopy with biopsies in the presence of normal mucosa to rule out microscopic colitis.

Furthermore, our patient reported relatively mild symptoms of diarrhoea which were present 3 years before referral to the Department of Gastroenterology. This may suggest that breast cancer metastases in the colon can be present rather silent clinically and that the medical oncologist and the gastroenterologist should be aware of the possibility of gastrointestinal metastases.

To differentiate primary from metastatic tumours involving the GI tract, histopathological comparison of mammary and GI specimens is mandatory including oestrogen receptor staining (Figure 2). Only two reports described the coexistence of breast cancer metastases and adenocarcinoma in the GI tract [6, 7].

The prognosis is usually poor in patients with metastatic breast cancer to the GI tract as a retrospective study of 73 patients found a median overall survival of 28 months [8]. Data on treatment is sparse, although it might consist of systemic treatment with chemotherapy or/and endocrine therapy, or surgical resection. Our patient was treated with Tamoxifen which resulted in disappearance of her diarrhoea.

## 4. Conclusion

GI metastases of breast carcinoma are rare and invasive lobular carcinoma metastasizes more frequently to the GI tract than ductal carcinoma. This is an important aspect in follow-up of these patients. Our case stresses the importance of considering breast cancer metastases as a potential cause of diarrhoea.

## Additional Points

GI metastases of breast carcinoma are rare and invasive lobular carcinoma metastasizes more frequently to the GI tract than ductal carcinoma. Endoscopic findings of breast cancer metastases to the colon might appear as an obstructing mass, Crohn's disease, or polyps or simulate linitis plastica of the colon. Our case emphasizes that endoscopic findings might be almost normal in patients with lobular breast cancer metastases to the colon. Further, GI complaints can be mild which suggests that the medical oncologist and the gastroenterologist should be aware of the possibility of gastrointestinal metastases and should perform diagnostic endoscopy including biopsies.
